# Emerging phenomena in nanophotonics

**DOI:** 10.1515/nanoph-2025-0171

**Published:** 2025-04-23

**Authors:** Donghyun Kim, Cheng-Wei Qiu, Yuri Kivshar, Dong-Il Yeom, Hong-Gyu Park

**Affiliations:** 26721 Yonsei University, Seoul, Republic of Korea; National University of Singapore, Singapore, Singapore; Australian National University, Canberra, Australia; Ajou University, Suwon, Republic of Korea; Seoul National University, Seoul, Republic of Korea

The 16th Pacific Rim Conference on Lasers and Electro-Optics (CLEO-PR 2024), held from August 4 to 9, 2024, in Incheon, Korea, presented groundbreaking advancements in nanophotonics, highlighting the rapid and dynamic evolution of the field. As a pivotal event for the global photonics community, CLEO-PR 2024 welcomed over 1,500 participants from 36 countries, fostering collaboration and knowledge exchange. With 156 organized sessions and more than 1,100 presentations, the conference underscored the transformative impact of interdisciplinary approaches and technological innovations on the field. This special issue features invited papers from selective presentations focusing on ultrafast and other emerging areas in nanophotonics.

We collected exciting papers including four reviews, one perspective, one letter, and 12 research articles. Specifically, “Programmable photonic unitary circuits for light computing” by Kim et al. [1]; “Exploring the frontier: nonlinear optics in low dimensional materials” by Adeshina and Kim [2]; “Emergent 2D van der Waals materials photonic sources” by Tang et al. [3]; “Stimulated Brillouin scattering in micro/nanophotonic waveguides and resonators” by Ren et al. [4]; “Deterministic generation and nanophotonic integration of 2D quantum emitters for advanced quantum photonic functionalities” by So [5]; “Harnessing in-plane optical anisotropy in WS_2_ through ReS_2_ crystal” by Kwon et al. [6]; “Frequency-comb-referenced multiwavelength interferometry for high-precision and high-speed 3D measurement in heterogeneous semiconductor packaging” by Park et al. [7]; “Nanoscale heat generation in a single Si nanowire” by Kim et al. [8]; “Visible transparency modulated cooling windows using pseudorandom dielectric multilayers” by Seo et al. [9]; “W-band frequency selective digital metasurface using active learning-based binary optimization” by Kim et al. [10]; “Electrodynamics of photo-carriers in multiferroic Eu_0.75_Y_0.25_MnO_3_” by Huang et al. [11]; “Polarization-independent narrowband photodetection with plasmon-induced thermoelectric effect in a hexagonal array of Au nanoholes” by Kim et al. [12]; “All-optical switch exploiting Fano resonance and subwavelength light confinement” by Saudan et al. [13]; “Efficient non-Hermitian wave-modulation protocol with a rapid parametric jump” by Shin et al. [14]; “Colloidal-quantum-dot nanolaser oscillating at a bound-state-in-the-continuum with planar surface topography for a high Q-factor” by Lee et al. [15]; “On-chip manipulation of trion drift in suspended WS_2_ monolayer at room temperature” by Choi et al. [16]; “Resonance modes in microstructured photonic waveguides: efficient and accurate computation based on AAA rational approximation” by Binkowski et al. [17]; “Dielectric permittivity extraction of MoS_2_ nanoribbons using THz nanoscopy” by Kelleher et al. [18] consist of the special issue.

Nanophotonics, the study of light–matter interactions on the nanoscale, has seen remarkable progress in recent years. Some key areas, particularly in ultrafast nanophotonics, include ultrafast light–matter interactions with ultrafast lasers to enable precise control of electron dynamics in nanomaterials and to explore new regimes of nonlinear optics, plasmonics and metasurfaces for extreme light confinement and real-time tunability for applications in ultrafast switching and beam steering, on-chip photonics & optical computing to implement ultrafast nanophotonic components and emerging materials, quantum nanophotonics with single-photon emitters in solid-state platforms and ultrafast control of quantum states, and finally, topological and non-Hermitian photonics with gain/loss engineering to offer new ways to control ultrafast light behavior. The future of nanophotonics as an area will be shaped by emerging materials, advanced fabrication techniques, and new physical insights. We hope that this feature issue will provide insights on the exciting frontiers that lie ahead in this rapidly evolving field of nanophotonics.
